# Left Atrial Hemodynamics and Clinical Utility in Heart Failure

**DOI:** 10.31083/j.rcm2509325

**Published:** 2024-09-11

**Authors:** Chang- Yi Lin, Shu- I Lin, Ying- Hsiang Lee, Chun- Yen Chen

**Affiliations:** ^1^Cardiovascular Division, Department of Internal Medicine, Mackay Memorial Hospital, Mackay Medical College, 104217 New Taipei City, Taiwan; ^2^Department of Nursing, Mackay Junior College of Medicine, Nursing and Management, 104217 New Taipei City, Taiwan

**Keywords:** left atrial pressure, heart failure, interatrial shunt devices

## Abstract

Comprehensive knowledge of the left atrium (LA) and its pathophysiology has emerged as an important clinical and research focus in the heart failure (HF) arena. Although studies on HF focusing on investigating left ventricular remodeling are numerous, those on atrial structural and functional changes have received comparatively less attention. Studies on LA remodeling have recently received increasing attention, and LA pressure (LAP) has become a novel target for advanced monitoring and is a potential therapeutic approach for treating HF. Various devices specifically designed for the direct measurement of LAP have been developed to optimize HF treatment by reducing LAP. This review focuses on LA hemodynamic monitoring and effective LAP decompression.

## 1. Introduction 

The pharmacological treatment of patients with heart failure and reduced ejection fraction (HFrEF) has revealed benefits in cardiac remodeling, symptom alleviation, and improvement in cardiac function and prognosis [1]. Furthermore, the treatment for heart failure with preserved ejection fraction (HFpEF) has shown positive outcomes with the use of sodium-glucose co-transporter-2 inhibitors (SGLT2i) [2, 3]. Patients with HFpEF who experience dyspnea during exertion may result from abrupt pulmonary congestion due to increased left ventricular and left atrial pressure (LAP) [4]. Patients with mitral stenosis (MS) and concomitant small congenital atrial septal defects (Lutembacher syndrome) exhibit fewer symptoms and better outcomes than those with isolated MS [5]. Therefore, understanding the role of left atrial structure and function in the pathophysiology of heart failure (HF) is critical. Traditionally, studies on the hemodynamics of HF have focused on the left ventricular structure and function. The left atrium (LA) is a passive chamber that carries blood into the left ventricle (LV); however, recent developments have highlighted the significance of LA function and structure as novel contributors to clinical outcomes in patients with HF [6, 7]. Clinicians’ interest in LAP often revolves around its preload contribution to the cardiac output. Increased LAP may be due to preexisting LV systolic and/or diastolic dysfunction and mitral and/or aortic valve insufficiency, and acute increases in LAP are observed in critical conditions, such as myocardial ischemia, stress-induced cardiomyopathies, and volume overload states [8]. Increased LAP has important effects on gas exchange, pulmonary hemodynamics, and right ventricular function [9, 10]; increased LAP levels are associated with LA remodeling [11]. Based on these studies, we found that a direct approach to unloading the LA (such as creating an iatrogenic interatrial shunt) has been suggested to improve HF-related symptoms and outcomes [12]. Therefore, optimizing clinically significant LAP decompression may be a favorable management strategy for HF. We discuss the hemodynamics and efficacy of the LA decompression device (LADd) for treating HF in this review.

## 2. Basic Structure, Function, and Physiology of the Left Atrium 

### 2.1 Anatomy

The LA is positioned at the posterior aspect of the four cardiac chambers [13]. Following the direction of blood flow, the LA starts at the junctions of the pulmonary veins (PVs) and ends at the fibrofatty tissue plane, precisely at the atrioventricular junction of the mitral orifice [13]. The five muscular walls of the LA are described as superior, posterior, left lateral, septal (or medial), and anterior, based on their location, as suggested by McAlpine [14]. The normal diameter of the LA is sex-dependent, defined as <4.1 cm for men and <3.9 cm for women, as measured using M-mode echocardiography. Measurements were taken in the parasternal long-axis view from the posterior aortic wall to the posterior left atrial wall during end-ventricular systole [15]. The LA volume (LAV) index (LAVI) is used to assess LA size in relation to an individual’s body surface area, calculated using the Mosteller formula. Khan *et al*. [16] reported a normal LAVI of 21–52 mL/m^2^, measured using cardiovascular magnetic resonance (CMR) in healthy subjects in the United States. Volumetric analysis of LA phasic function was derived from measurements of the maximum (at LV end-systole), minimum (at LV end-diastole), and LAV immediately before atrial contraction. Maximum LAV (LAV_max_) has been used in randomized clinical trials [17, 18] and in the UK Biobank CMR study [19]. The Copenhagen City Heart Study showed that the minimum LAVI (LAVI_min_) was an independent predictor of incidence of HF in low-risk subjects [20]. LAVI_min_ also tends to have stronger additional prognostic value than LAVI_max_ [21, 22], and LAVI_min_ may also better reflect LV end-diastolic pressure [23]. When the mitral valve opens during diastole, the LA is continuously exposed to LV pressure. LAVI_min_ can thus better reflect LV filling pressure and pulmonary artery wedge pressure (PAWP). Despite these advantages, LAVI might be unreliable when patients receive HF therapy because LA enlargement persists despite HF therapy normalizes LV filling pressure. Therefore, LAVI should be used in combination with other indices, such as LA strain when evaluating LAP [24, 25].

The PVs enter the posterior part of the LA, with the left veins positioned superior to the right veins [13]. Conventionally, the four PVs transport blood from the lungs into the LA separately but only account for 70% of the population [26]. Approximately 12%–25% of individuals have funnel-like common veins, with either the right or left PVs entering through a single ostium [26], and no valves were observed between the PVs and the LA.

### 2.2 Function and Physiology

The LA receives oxygen-rich blood from the lungs via the PVs and pumps it into the LV. Barbier *et al*. [27] outlined three distinct phases of LA function: a reservoir during systole, a conduit in early diastole, and a booster pump in late diastole. During the systolic phase, the mitral valve closes, and the LA begins to expand and acts as a reservoir to accumulate blood from the PVs. This phase signifies LA relaxation and compliance modulated by LV systolic function through the descent of the LV base. During early LV diastole, the mitral valve opens, blood floods into the LV, and the LA transforms into a conduit that facilitates the passage of blood between the PVs and LV. This conduit function relies on the LV diastolic function and includes both the suction force, which depends on LV relaxation, and LV chamber stiffness. The booster function of the LA relies on intrinsic LA contractility, LV end-diastolic compliance, and pressure. The lack of an LA booster is associated with a 20–30% reduction in LV stroke volume [28].

### 2.3 Measurement of LAP

LAP varies throughout the cardiac cycle, and this fluctuation is caused by the interaction between the incoming flow from the PVs into the LA and the ongoing flow from the LA into the LV. Although LV end-diastolic pressure (LVEDP) and mean LAP are often used interchangeably, they convey distinct information. LVEDP provides information on LV operating compliance and is the closest estimate of LV preload as a surrogate for LV end-diastolic volume (LVEDV). In contrast, the mean LAP integrates atrial pressure tracing throughout systole and diastole, thus reflecting the impact of pulmonary venous circulation on right ventricular performance. The presence of large “V” waves, observed in conditions such as reduced LA compliance, severe mitral regurgitation, or atrial fibrillation, can lead to significant differences between the mean LAP and LVEDP [29]. Measuring mean LAP facilitates the differentiation of post-capillary pulmonary hypertension (PH) [29, 30].

### 2.4 Invasive Method: Pulmonary Artery Wedge Pressure and Direct Measurement of LAP

The standard way to measure PAWP is to evaluate patients who take chronic medications in a non-fasting state, without sedation, and in the supine position, with a 7Fr fluid-filled Swan-Ganz catheter inserted into the pulmonary artery through the internal jugular vein. Normal individuals show, on average, a resting right atrial pressure (RAP) of 4 mmHg [1–5 mmHg], a peak RAP of 5 mmHg [4–7 mmHg], and an RAP/cardiac output (CO) slope of 0.32 mmHg/L/min. Abnormal references are defined by values of the >97.5th percentile: resting RAP >7 mmHg, peak RAP >12 mmHg, and RAP/CO slope ≥1.30 mmHg/L/min. Patients with HFpEF often present higher peak RAP and RAP/CO slopes than patients with pulmonary arterial hypertension (PAH) (20 mmHg vs 12 mmHg and 3.47 mmHg/L/min vs 1.90 mmHg/L/min, *p* < 0.05) [31]; therefore, direct measurements can accurately reflect the LAP. Faisal Fa’ak *et al*. [32] introduced a straightforward and secure technique using a one-catheter strategy with a TIG catheter (5Fr tiger-shaped Optitorque Diagnostic Catheter; Terumo Interventional Systems, Somerset, NJ, USA) that traversed the aortic valve and crossed the mitral valve retrogradely.

### 2.5 Noninvasive Methods: Echocardiography and Doppler Techniques

The 2016 American Society of Echocardiography and the European Association of Cardiovascular Imaging guidelines suggest estimating the mean LAP through a Doppler assessment of diastolic blood flow between the LA and LV (mitral E to A wave ratio), which involves tissue Doppler imaging (TDI) of the mitral annulus, the tricuspid regurgitant flow velocity, and LA volumes [24]. However, its diagnostic accuracy is limited in patients with unexplained dyspnea and/or PH, with low sensitivity for detecting diastolic dysfunction [33]. Therefore, using a single parameter to evaluate LAP should be avoided [34]. As a noninvasive, quick bedside screening tool, the “rule of 8’s” is suggested: a lateral E/e’ of >8 [35] and a lateral e’ of ≤8 cm/s [36].

In recent years, two novel noninvasive methods have emerged: LA strain and the LA expansion index (LAEI). LA strain is measured using speckle tracking in the non-foreshortened apical-4-chamber view of the LA, to assess LA function and stiffness [37]. The cutoff values for an LA reservoir strain of <18% (area under the curve [AUC]: 0.76) and an LA pump strain of <8% (AUC: 0.77) are used to detect increased LV filling pressure (defined as PAWP >12 mmHg or LVEDP >16 mmHg) [38]. The LAEI represents the percentage of LA volume change over the cardiac cycle and is calculated using the following formula: (maximal LA volume × minimal LA volume) ×100%/minimal LA volume. Genovese *et al*. [39] found that the LAEI correlated logarithmically with pulmonary capillary wedge pressure (PCWP) in over 600 individuals from a cohort of patients with chronic cardiac disease.

### 2.6 Implantable Hemodynamic Monitoring Devices 

An implantable hemodynamic monitor (IHM) has been developed for outpatient HF management. The device (Chronicle, Medtronic Inc., Minneapolis, MN, USA) has been implanted transvenously and continuously to measure and store hemodynamic information [40]. IHM devices that directly measure pulmonary artery pressure (PAP) have shown efficacy in reducing hospitalizations for HF [41], although evidence that supports a reduction in overall mortality remains limited [42].

## 3. Treatments that Target Mean LAP

### 3.1 Mean LAP as a Treatment Target in Heart Failure

In the field of HF treatment, several pharmacological therapies have decreased mortality in patients with HFrEF, including beta-blockers [43], angiotensin-converting enzyme inhibitors/angiotensin II receptor blockers [44, 45], and mineralocorticoid/aldosterone receptor antagonists [46]. Recent advancements have introduced novel pharmaceutical agents, such as neprilysin inhibitors [47], SGLT2i [48] and ivabradine [49], which have been effective in treating HFrEF. Cardiac resynchronization therapy, implantable cardioverter defibrillators, coronary artery bypass surgery, transcatheter or surgical aortic valve implantation, and transcatheter edge-to-edge repair have benefitted select patients with HFrEF and, therefore, have been included in established guidelines as non-pharmacological-treatments for patients with HFrEF [1, 50]. Contrary to the vigorous advances in HFrEF treatment, the treatment landscape for HFpEF has attained limited development over the past decade. The latest guidelines recommend only diuretics and SGLT2i as Class I pharmacological therapies [51]. Owing to the unmet need to treat HFpEF, device-based therapies have emerged as alternative approaches for targeting HF-related hemodynamic abnormalities that do not respond completely to pharmacological therapies.

Elevated LAP at rest or during exercise is associated with dyspnea, reduced exercise capacity, and unfavorable outcomes in patients with HFpEF [51, 52], and several medical therapies offer indirect benefits by decreasing LAP and mitigating LA remodeling [53, 54, 55]. Considering that elevated LAP levels are a pivotal mechanism underlying HF symptoms, device-based treatments aimed at unloading LAP have been proposed to improve HF-related symptoms and outcomes. One treatment involves accelerated pacing, and this concept is supported by prior clinical trials indicating that increasing heart rate (HR) in patients with pacemakers can improve functional capacity and reduce N terminal pro B type natriuretic peptide (NT-proBNP) levels [56]. However, this result remains controversial, as some studies have suggested that increasing HR shortens the diastolic LV filling time and induces LAP elevation [57, 58]. Different outcomes may arise from the distinct HF phenotypes [59]; for example, patients with advanced HFpEF typically develop chronotropic incompetence. A higher pacing rate has the potential benefit of normalizing LAP and improving respiratory conditions [60]. The PACE HFpEF trial is an ongoing single-center prospective pilot study in which pacemakers are being implanted in patients with HFpEF without prior indications for pacemakers, and holistic pacing are administered via the His or Bachmann bundle leads [61]. This study aims to test the hypothesis that moderately accelerated pacing can normalize elevated LAP or LVEDP, thereby improving symptoms and enhancing physical function in patients with HFpEF. In addition to the pacemaker pacing approach, emerging studies are testing therapeutic strategies that aim to directly unload the LAP through structural intervention[62, 63].

### 3.2 Directly Unloading Mean LAP to Treat HF

The concept of creating an interatrial shunt as a potential therapy for patients with HF has been supported by observations in Lutembacher’s syndrome, where patients with concomitant atrial septal defects and MS tend to exhibit improved symptoms and outcomes than those with MS alone [64, 65]. The postulated mechanism suggests that the presence of a left-to-right shunt alleviates the hemodynamic burden on the LA in patients with MS, leading to percutaneous balloon septostomy.

The concept of creating an iatrogenic interatrial shunt was initially applied to patients undergoing extracorporeal membrane oxygenation (ECMO) for refractory pulmonary edema-related hypoxemia [66]. The arterial cannula in venoarterial ECMO can increase LV afterload, consequently increasing LV end-diastolic and LAP and exacerbating LV failure and pulmonary edema. Percutaneous balloon septostomy has successfully achieved LA decompression in 50–66% of patients and has resulted in left atrial pressure reduction and clinical improvement in >95% of patients [66, 67]. Atrial septostomy-based cannulation as an LV venting strategy has also been developed to achieve a more effective reduction in LAP, thereby indirectly reducing LVEDP. The procedure involves percutaneous balloon septostomy and percutaneous insertion of a left atrial drainage catheter. Retrospective data suggest that LV venting may be efficacious in improving short-term outcomes, particularly when initiated early, and may be associated with lower hospital mortality rates [68]. Recently, the EARLY-UNLOAD study, a randomized controlled trial aimed at evaluating the optimal timing for LA unloading when using venoarterial ECMO, reported that at 30 days, there was no significant difference in all-cause mortality between patients undergoing routine transeptal left atrial canulation within 12 hours and the conventional group, which allowed for rescue venting if needed [69]. The DanGer Shock trial showed that unloading the LV by a microaxial flow pump with standard care was used in patients with ST-segment elevation myocardial infarction-related cardiogenic shock, which led to a lower risk of death from any cause but a higher incidence of adverse events at 180 days than standard care alone [70]. This result may be explained by the diverse etiologies of cardiogenic shock. Furthermore, LV unloading using an intra-aortic balloon pump or microaxial flow pump is crucial to maximize myocardial recovery in patients experiencing cardiogenic shock related to acute myocardial infarction (AMI). However, LV venting via transseptal LA cannulation primarily aims to reduce pulmonary congestion and enhance gas exchange, resulting in a relatively lower benefit in AMI-related cardiogenic shock than in acute decompensated HF-related cardiogenic shock. This may explain the neutral outcomes of the EARLY-UNLOAD trial that enrolled a considerable number of patients with AMI-related cardiogenic shock [71].

Balloon septostomy only provides transient therapeutic effects, as long-term patency is rarely achieved [67]. Durable devices are required for patients with chronic HF; hence, delicate percutaneous device-based shunt therapies have emerged as novel therapeutic strategies.

### 3.3 Interatrial Shunt Devices

In chronic HF, elevated LAP and pulmonary pressure are the key determinants of exercise limitation. Interatrial shunt devices create a permanently controlled left-to-right shunt to relieve LAP, particularly in patients who exhibit increased LAP during exercise but not at rest. The InterAtrial Shunt Device (IASD; Corvia Medical), which is a self-expanding metal stent with a double-disc shape and a central opening of 8 mm, is the first and most extensively studied shunt device. The IASD system is delivered percutaneously through the femoral vein for implantation across the interatrial septum, thereby establishing a pressure-dependent left-to-right flow.

The REDUCE LAP-HF trial was an open-label, single-arm, phase 1 study that evaluated 68 patients with symptomatic HF with an ejection fraction (EF) of ≥40% treated with IASD [62]. At six months, 52% of the patients experienced a reduction in PCWP at rest, whereas 58% showed a lower PCWP during exertion. The mean exercise PCWP significantly decreased from 32 mmHg at baseline to 29 mmHg at 6 months. Subsequently, the REDUCE LAP-HF I was a randomized, sham-controlled trial that involved 94 patients with HF with an EF of ≥40% [63]. At 1 month, the IASD group achieved a significantly greater PCWP reduction than the sham control group. No periprocedural or cardiovascular adverse events were reported at the 1-month follow-up, and no significant differences in major adverse cardiac, cerebrovascular, or renal events were identified at the 1-year follow-up [72]. The phase 3 REDUCE LAP-HF II trial randomized 626 patients with HF with an EF of ≥40%, an exercise PCWP of >25 mmHg, and at least 5 mmHg higher than the right atrium (RA) pressure to either IASD or a sham procedure. The results showed no difference between the two groups in terms of the primary endpoint, which was a hierarchical composite of cardiovascular mortality or nonfatal ischemic stroke at 12 months, the rate of total HF events up to 24 months, nor the change in the Kansas City Cardiomyopathy Questionnaire (KCCQ) score at 12 months [73]. However, the impact of IASD treatment on HF events varied across different pre-specified subgroups. A pulmonary artery systolic pressure of <70 mmHg at 20 W of submaximal exercise, a right atrial volume ≤29.7 mL/m^2^, and female sex were identified as factors that were associated with benefits from the atrial shunt device.

An atrial shunt reduces the PCWP by redistributing blood flow to the right side of the heart, resulting in an approximately 25% increase in pulmonary blood flow. Although increased pulmonary blood flow yields favorable short-term effects, sustained elevation can precipitate PH, and cause right ventricular dysfunction and clinical RV failure. The REDUCE LAP-HF II trial excluded patients with severe pulmonary vascular disease (PVD), which is characterized by right ventricular dysfunction, right atrial pressure >14 mmHg, and resting pulmonary vascular resistance (PVR) >3.5 wood units (WUs). However, latent PVD, characterized by a PVR of ≥1.74 WU at peak exercise, may persist. Patients with latent PVD may derive fewer benefits from atrial shunting devices, owing to a minimized or even reversed LA-to-RA pressure gradient. This reversal occurs because the increased pulmonary blood flow leads to right heart congestion after atrial shunting. A post hoc analysis from the REDUCE LAP-HF II trial revealed that patients with latent PVD experienced worse outcomes and symptoms, whereas those without latent PVD benefited from shunt-mediated LA unloading [74]. The post hoc analysis of REDUCE LAP-HF II trial with over 2 years of follow-up showed IASD led to reverse remodeling of left-sided chambers while expanding right-sided chambers in HFpEF patients and did not significantly impact RV systolic function compared with sham. The responders (no latent PVD and no cardiac rhythm management device) had more favorable changes in cardiac structure and function compared with non-responders [75]. The ongoing RESPONDER-HF trial, a randomized, sham-controlled, double-blinded trial, is designed to evaluate the efficacy and safety of IASD in patients with chronic HF, LVEF of ≥40%, and an absence of latent PVD (ClinicalTrials.gov Identifier: NCT05425459, https://clinicaltrials.gov/ct2/show/NCT05425459).

Several new shunt devices have been developed and investigated (Table 1, Ref. [72, 73, 76, 77, 78, 79, 80]), and initial first-in-human or pilot studies have been completed and have primarily reported improved PCWP, New York Heart Association class, or 6-minute walking distance, with an acceptable patency rate and safety profile. However, further trials are required to assess the clinical outcomes of the new devices.

**Table 1.** **Devices directly unloading left atrium pressure**.

Device	Study	Target group	Main findings
IASD (Corvia Medical)	REDUCE LAP-HF I & II, RCT, double-blinded, sham-controlled [72, 73]	HFpEF and HFmrEF	Exercise PCWP ↓
			No difference in composite primary endpoint
V-Wave Gen2 (V-Wave Ltd)	Pilot, single arm [76]	HFrEF and HFpEF	NYHA class ↓
			KCCQ ↑
Occlutech AFR (Occlutech AG)	Pilot, single arm [77, 78]	HFrEF and HFpEF	NYHA class, rest PCWP, NT-proBNP ↓
			KCCQ, 6MWT ↑
Transcatheter atrial shunt system (Edwards Lifesciences)	First-in-human study [79]	HFrEF and HFpEF	NYHA class ↓
			Rest PCWP ↓
D-Shant (Wuhan Vickor Medical Technology Co., Ltd.)	First-in-human study [80]	HFrEF and HFpEF	LV diameter ↓
			LV volume ↓
			LAP, PCWP ↓
			NYHA class ↓
			KCCQ, 6MWT ↑

Abbreviation: RCT, randomized controlled trial; HFpEF, heart failure with preserved ejection fraction; HFmrEF, heart failure with mid-range ejection fraction; HFrEF, heart failure with reduced ejection fraction; PCWP, pulmonary capillary wedge pressure; NYHA, New York Heart Association; KCCQ, Kansas City Cardiomyopathy Questionnaire; NT-proBNP, N terminal pro B type natriuretic peptide; 6MWT, 6 minute walk test; LV, left ventricule; LAP, left atrium pressure; AFR, atrial flow regulator; IASD, InterAtrial Shunt Device; ↓, decrease; ↑, increase.

## 4. Conclusions 

Elevated LAP levels may drive the clinical symptoms of HF and are associated with poor outcomes in patients with HF. Accurate assessment of LAP using a pulmonary artery catheter, echocardiographic Doppler, and two-dimensional (2D) techniques may provide clinical benefits for the treatment of HF. Evidence supporting therapeutic interventions to unload LAP, resulting in reverse LA remodeling, has increased since the initiation of HF therapy; however, the effect of this improvement in LA function on clinical outcomes remains unclear. Evaluation of targeted therapies to reduce LAP responses in various HF phenotypes may redefine patient risk stratification. Furthermore, identifying structural and biological HF-related abnormalities that may not be suitable for reducing LAP using pharmacological therapies is important when device-based therapies (such as IASD) are used as an alternative approach.
